# Restoration of NK Cell Cytotoxic Function With Elotuzumab and Daratumumab Promotes Elimination of Circulating Plasma Cells in Patients With SLE

**DOI:** 10.3389/fimmu.2021.645478

**Published:** 2021-03-22

**Authors:** Morgane Humbel, Florence Bellanger, Natalia Fluder, Alice Horisberger, Madeleine Suffiotti, Craig Fenwick, Camillo Ribi, Denis Comte

**Affiliations:** Service of Immunology and Allergy, Lausanne University Hospital, University of Lausanne, Lausanne, Switzerland

**Keywords:** systemic lupus erythematosus (SLE), SLAMF, CD38, elotuzumab, daratumumab, NK cells, CD150/SLAMF1 receptor, CD319/SLAMF7/CS1

## Abstract

Systemic lupus erythematosus (SLE) is a multisystem autoimmune disease characterized by multiple cellular and molecular dysfunctions of the innate and adaptive immunity. Cytotoxic function of NK cells is compromised in patients with SLE. Herein, we characterized the phenotypic alterations of SLE NK cells in a comprehensive manner to further delineate the mechanisms underlying the cytotoxic dysfunction of SLE NK cells and identify novel potential therapeutic targets. Therefore, we examined PBMC from SLE patients and matched healthy controls by single-cell mass cytometry to assess the phenotype of NK cells. In addition, we evaluated the cell function of NK cells (degranulation and cytokine production) and the killing of B cell subpopulations in a B cell-NK cell *in vitro* co-culture model. We found that SLE NK cells expressed higher levels of CD38 and were not able to adequately upregulate SLAMF1 and SLAMF7 following activation. In addition, ligation of SLAMF7 with elotuzumab or of CD38 with daratumumab on SLE NK cells enhanced degranulation of both healthy and SLE NK cells and primed them to kill circulating plasma cells in an *in vitro* co-culture system. Overall, our data indicated that dysregulated expression of CD38, SLAMF1 and SLAMF7 on SLE NK cells is associated with an altered interplay between SLE NK cells and plasma cells, thus suggesting their contribution to the accumulation of (auto)antibody producing cells. Accordingly, targeting SLAMF7 and CD38 may represent novel therapeutic approaches in SLE by enhancing NK cell function and promoting elimination of circulating plasma cell.

## Introduction

Systemic lupus erythematosus (SLE) is a multisystemic autoimmune disease that mainly affects women of childbearing age (1, 2). The pathogenesis remains elusive but includes alterations of the immune system leading to the production of autoreactive cells, autoantibodies and the formation of immune complexes that ultimately damage organs (1, 3). Although important progress was made over the last decades toward the development of new treatments, management of SLE still relies on the use of corticosteroids and immunosuppressive agents that non-specifically target immune cells. Despite the well-established importance of autoreactive B cells and autoantibody production in the pathogenesis of the disease (1), treatments based on B cell depletion have only been moderately successful so far (4). In this context, understanding the role of other immune cells involved in the pathogenesis of SLE and their link with antibody-producing cells is taking a center stage in the development of new therapies. Among the various cellular abnormalities that characterize SLE, Natural Killer (NK) cells’ dysfunction has been supported by various studies (5–8). NK cells are innate lymphocytes that play a pivotal role in the immune surveillance (9), through the recognition of healthy cells and the elimination of damaged or infected cells. NK cells from SLE patients are reduced in number in the peripheral blood, show impaired cytokine production upon stimulation, reduced cytotoxicity, and defective antibody-dependent cellular cytotoxicity (5). However, their exact role in the pathogenesis of lupus remains elusive.

In the present study, we used single-cell mass cytometry to perform a comprehensive phenotypic analysis of healthy and SLE NK cells. We sought to identify how these alterations are linked to the altered function of SLE NK cells and might represent therapeutic options to treat SLE.

## Materials and Methods

### SLE Patients and Controls

SLE patients (N=44) were diagnosed according to the American College of Rheumatology classification criteria and/or the Systemic Lupus International Collaborating Clinics (SLICC) criteria (10, 11), and were recruited from the Division of Immunology and Allergy at Centre Hospitalier Universitaire Vaudois (CHUV). All patients and controls were included in the Swiss Systemic Lupus Erythematosus Cohort Study (SSCS) (12). Characteristics of the SLE patients included in this study are provided in **Table 1**.

**Table 1:** Demographic characteristics of SLE patients (N=44) included in the study.

Characteristic	Value
Age, years	
Median	46
Range	24-73
Sex	
Female	37
Male	7
Ethnicity	
Caucasian	40
Asian	3
Hispanic	1
SLE disease activity	
Inactive (0-3)	21
Moderate (4-10)	15
Active (>10)	8
Treatments	
Naïve	7
Hydroxychloroquine only	11
Other immunomodulatory drugs	25

Age-, sex-, and ethnicity-matched healthy individuals were chosen as controls. Disease activity score was measured using the SLE Disease Activity Index (SLEDAI) scoring system. We categorized patients into three groups of disease activity: inactive (SLEDAI 0-3), moderate (SLEDAI 4-10) and active (SLEDAI >10).

### Cell Isolation

Peripheral blood mononuclear cells (PBMC) were enriched by density gradient centrifugation (FICOLL 400, Merck, Switzerland). PBMC were cryopreserved in liquid nitrogen.

### Cell Culture

Cells were cultured in RPMI (Gibco; Life Technologies) containing 10% heat-inactivated FBS (Institut de Biotechnologies Jacques Boy), 100 IU/ml penicillin and 100 μg/ml streptomycin (Bio Concept), hereafter referred to as complete RPMI (cRPMI).

### Antibodies

A complete list of mass cytometry, flow cytometry and purified antibodies is provided in the **Supplementary Table 1**.

Some antibodies for the mass cytometry assay were conjugated in our facility (MaxPar^®^ X8 multimetal labeling kit, Fludigm). Briefly, the MaxPar^®^ polymer is loaded with the metal, and then the antibody is partially denatured to allow its conjugation to the polymer. Finally, the metal bound polymer is conjugated to the antibody.

### Mass Cytometry

Cryopreserved PBMC from SLE patients and matched healthy controls were thawed, resuspended in cRPMI, stimulated with cytokines or left unstimulated as mentioned in the figures. Cells were stained for live/dead with cisplatin 50 µg (5min, room temperature (RT)), barcoded with CD45-metal conjugated antibodies (20min, RT) and then pooled. Next, cells were incubated with metal conjugated antibody mix (20min, RT). Cells were washed and fixed with 2.4% paraformaldehyde (10 min; RT). Labeled samples were acquired on a Helios Cytof System (Fluidigm). Flow cytometry standard (FCS) files were normalized to EQ Four Element calibration beads using CyTOF software. FCS files were debarcoded using Cytobank (Beckman Coulter).

### Mass Cytometry Data Analysis

Manual gating of FCS files was performed using FlowJo™ Software version 10.2 (Becton, Dickinson and Company; 2019). Data analysis was performed using R software (version 3.5.1.). Manually gated cell populations were imported into R environment and single cell expression data were transformed using hyperbolic inverse sine (with cofactor 5) (13). Dimensionality reduction and 2-dimensional visualization were done using the Barnes-Hut implementation of t-stochastic neighboring embedding algorithm (Rtsne package). Unsupervised clustering analysis on cell populations were performed using self-organizing map in combination with consensus clustering (FlowSOM package) in order to define 4 different clusters.

For the analysis of NK cells, we merged two experiments designed with two different panels using CytofMerge (14) with default settings. The CytofMerge methodology is based on the k-nearest neighbor algorithm and a set of common markers in order to impute the value of missing markers by taking the median values of from the k most similar cells.

### NK Cells Cytokine Production and Degranulation

PBMCs were thawed and resuspended in cRPMI. For evaluation of degranulation, NK cells were stimulated with IL-15 (50ng/ml), IL-18 (50ng/ml) or a combination of IL-2 (50ng/ml) and IL-12 (20ng/ml). For evaluation of NK cell activation with monoclonal antibodies cells were resuspended in cRPMI with IL-15 (1ng/ml). Cells were then stimulated with or without cytokines (IL-2 and IL-12, 50ng/ml and 20ng/ml respectively) with the following antibodies: SLAMF1 A12 (5µg/ml), SLAMF7 162.1 (5µg/ml), elotuzumab (0.1µg/ml), daratumumab (1µg/ml), elotuzumab and daratumumab (0.1µg/ml and 1µg/ml respectively) and incubated for either 6 or 18 hours at 37°C. BD GolgiPlug™, BD GolgiStop™ and CD107a-PE were added 6h before readout.

After incubation, cells were stained with Live/Dead Aqua and cell surface antibodies:CD3-BUV737, CD4-PB, CD8-BV605, CD19-FITC, CD56-BUV395. After permeabilization with BD Cytofix/Cytoperm™ kit, cells were stained with IFNγ-AF700, TNFα-APC. Finally, cells were fixed in BD CellFIX™ and stored at 4°C until data acquisition on LSR Fortessa™ (BD Bioscience).

### NK and B Cells Co-Culture

PBMC cells from HC were thawed and sequential positive selection of CD19+ and CD56+ cells was performed (human microbeads, Miltenyi positive selection kits) using the AutoMACS® ProSeparator (Miltenyi Biotec). B cells were stained with CFSE (LifeTech). All cells were resuspended in cRPMI with IL-15 (1ng/ml).

NK cells were incubated for 30minutes at 37°C with the following stimulation conditions: unstimulated, SLAMF1 A12 (5µg/ml), SLAMF7 162.1 (5µg/ml), elotuzumab (0.1µg/ml), daratumumab (1µg/ml), elotuzumab and daratumumab (0.1µg/ml and 1µg/ml respectively). After incubation NK cells were washed and B cells were added (in cRPMI with IL-15) at a 2:1 ratio (NK min 500’000 cells, max 1Mio; B cells min 250’000, max 500’000 cells) and incubated for 5.30hours. After incubation cells were washed and stained with Live/Dead Aqua, CD56-BUV395, CD20-PB, CD21-AF700, CD27-PeCy7, CD38-ECD, SLAMF7-PE. Finally, cells were fixed in CellFIX™ and stored at 4°C until data acquisition on a LSR Fortessa™.

### Depletion Assay

PBMCs were thawed and CD3 negative cells were isolated (EasySep™ Human CD3 Positive Selection Kit II, StemCell Technologies). Cells were resuspended in cRPMI with IL-15 (1ng/ml), and the following stimulation conditions were added: not stimulated, SLAMF1 (5µg/ml) with cytokines (IL-2 (50ng/ml) and IL-12 (20ng/ml)), elotuzumab (0.1µg/ml) with cytokines, daratumumab (1µg/ml) with cytokines and HLA-DR (0.005µg/ml) with cytokines. CD3 negative cells were then incubated for either 6 or 18 hours at 37°C. After incubation, cells were stained extracellularly with Live/Dead Aqua, CD3-BUV737, CD19-FITC, CD20-PB, CD27-AF700, CD38-ECD, CD56-BUV395, SLAMF7-PE. Cells were then washed in annexin buffer (10X Annexin V Buffer, BD Pharmingen) and stained with Annexin V-APC. Cells were stored at 4°C until data acquisition on LSR Fortessa™, for maximum 2h.

### Statistics

Statistical analysis were performed using GraphPad Prism (version 8). Specifications of tests exploited and sample size for each experiment are mentioned in the figure descriptions. In a general manner, Mann-Whitney test was used for comparison between two groups with non-normal distribution (normality was assessed with Shapiro-Wilk test). Kruskal-Wallis test was used for the comparison of multiple groups with non-normal distribution and p-values were adjusted for multiple tests using Dunn’s method. One-way ANOVA was used for the comparison of multiple groups with normal distribution and p-values were adjusted for multiple tests using Sidak’s method. Two-way ANOVA was exploited for the comparison of multiple groups and p-values were adjusted for multiple tests using Sidak’s method. Two-way ANOVA was exploited for multiple comparisons within a group and p-values were adjusted for multiple tests using Tukey’s method. A p-value lower than 0.05 was considered significant.

### Study Approval

Informed written consent was obtained from all participants prior to inclusion and the study was approved by the Institutional Review Board (SwissEthics 2017-01434), in compliance with the Declaration of Helsinki.

## Results

### NK Cells Are Reduced in Numbers and Their Function Is Impaired in SLE Patients

**Figure 1:**
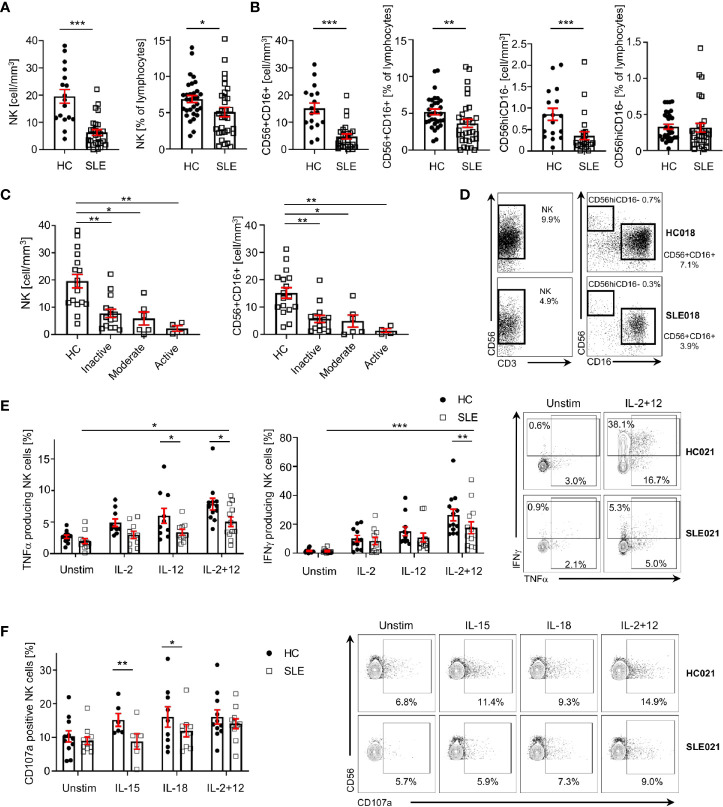
NK cells are decreased and dysfunctional in patients with SLE. **(A)** Total NK cells and **(B)** CD56+CD16+ and CD56hiCD16- NK subpopulations in SLE patients and HC are shown as absolute number (HC=17, SLE=27; Mann-Whitney Test) and percentage of total lymphocytes (HC=31, SLE=31; Mann-Whitney Test). **(C)** Total NK cells and CD56+CD16+ absolute number according to SLE disease activity (HC=17, Inactive=15, Moderate=6, Active=4; Kruskal-Wallis Test with Dunn’s multiple comparison test). **(D)** Representative dot-plot of NK cells (left) and subpopulations (right) staining gated on live CD45+CD14-CD7+CD20-CD19- cells. The percentages of NK, CD56+CD16+ and CD56hiCD16- refer to % of total lymphocyte count. **(E)** Cumulative results and representative dot-plot showing NK cell cytokines production in SLE and HC after overnight stimulation (IFNγ HC=12, SLE=12; TNFα HC=13, SLE=13; mixed-effects analysis and two-way ANOVA with Sidak’s multiple comparison test). **(F)** Cumulative results and dot-plot showing NK cell degranulation (CD107a+ cells) after overnight stimulation in SLE and HC (HC=11, SLE=11; two-way ANOVA and Sidak’s multiple comparison test). Data represent mean ± SEM (*P < 0.05, **P < 0.01, ***P < 0.001). HC, healthy controls.

As previously reported, SLE patients display a significant decrease in the absolute numbers of NK cells compared to sex-, age- and ethnicity- matched healthy controls (HC; **Figures 1A, D** **)**. Percentage and/or absolute numbers of CD56+CD16+ and CD56hiCD16- NK cell subsets are reduced in SLE patients (**Figures 1B, D** **)**. Decreased NK cell numbers correlate with disease activity, as patients with higher disease activity display a more profound reduction in NK cell numbers compared to patients with inactive disease and HC (**Figure 1C**).

To examine the function of NK cells in SLE, we stimulated NK cells with a combination of IL-2 and IL-12, which promoted the production of IFNγ and TNFα by SLE NK cells (**Figure 1E**), although significantly less compared to HC (**Figure 1E**). In response to IL-15 and IL-18, the degranulation of SLE NK cells is impaired compared to HC, as illustrated by the reduced frequency of CD107a+ NK cells in SLE patients (**Figure 1F**). Stimulation with IL-2 and IL-12 provided a strong enough stimulation to activate SLE NK cells degranulation as effectively as in HC (**Figure 1F**). Collectively, our data indicates that SLE NK cells display impaired cytokine production and reduced degranulation in response to activation with different cytokines.

### Phenotypic Alterations of SLE NK Cells

We exploited single-cell mass cytometry to decipher the extracellular phenotype of SLE NK cells. Our panels include lineage markers for T cells, B cells, NK cells, monocytes and dendritic cells. The gating strategy is presented in **Supplementary Figure 1**. Various markers that characterize NK cells subpopulations, as well as markers that have been shown to be aberrantly expressed on other cell subsets in SLE were examined. These include CD25, CD38, PD-1, activation receptors (NKp46, NKG2D, DNAM-1), inhibitory receptor (NKG2A. KIR2DL, KIR3DL) and receptors belonging to the SLAMF family, including SLAMF1 (CD150), SLAMF2 (CD48), SLAMF3 (CD229), SLAMF4 (CD244, 2B4), SLAMF5 (CD84), SLAMF6 (CD353, NTB-A) and SLAMF7 (CD319, CRACC, CS-1).

**Figure 2:**
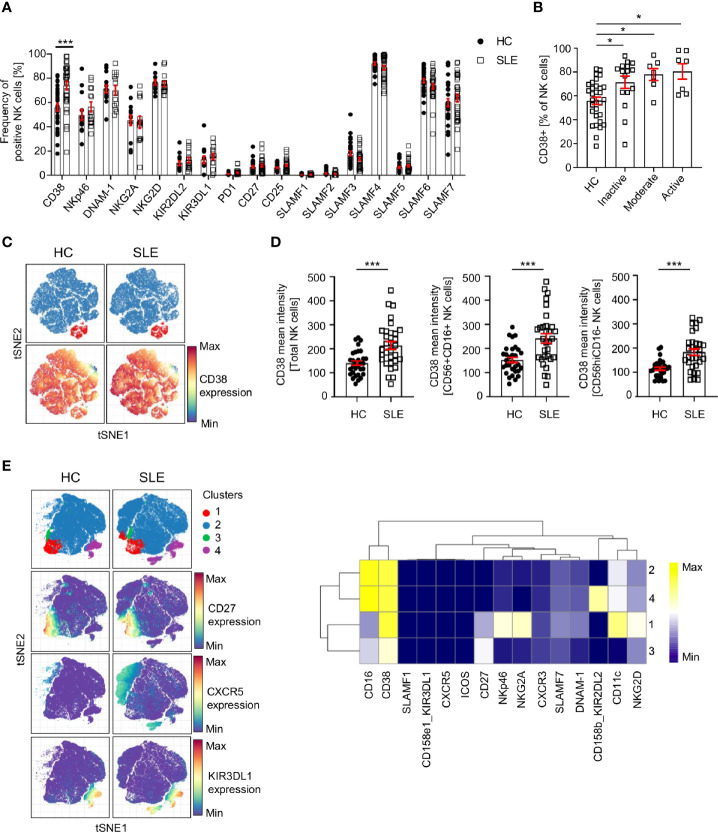
Analysis of NK cell surface markers in SLE patients and controls by single-cell mass cytometry. **(A)** Percentage of NK cells expressing the indicated cell surface markers in HC and SLE (HC=33, SLE=33; two-way ANOVA and Sidak’s multiple comparison test). **(B)** Frequency of CD38+ NK cells in SLE patients according to the SLE disease activity (HC=31, Inactive=16, Moderate=7, Active=7; Kruskal-Wallis Test with Dunn’s multiple comparison test). **(C)** Representative t-SNE analysis showing the expression of CD38 on SLE and HC NK cells (down-sample HC=30’000 cells, SLE=30’000 cells; blue: CD56+CD16+ NK cells; red: CD56CD16hi NK cells). **(D)** Cumulative results showing the expression (mean intensity) of CD38 on total NK, CD56+CD16+ and CD56hiCD16- NK cells (HC=31, SLE=31; Mann-Whitney test). **(E)** t-SNE analysis (down-sample HC=10’000 cells, SLE=10’000 cells) and heatmap showing NK cell clusters in HC and SLE patients. Data represent mean ± SEM (*P < 0.05, ***P < 0.001). HC, healthy controls.

Our data indicates that CD38 is expressed at a higher level in total SLE NK cells (CD3-CD14-CD7+CD19-CD56+) (**Figure 2A**; **Supplementary Figure 2A**). This difference is independent of disease activity (**Figure 2B**) and is also observed in CD56+CD16+ and CD56hiCD16- NK subsets (**Figures 2C, D** **)**. Similar results were found for treatment-naïve SLE patients (**Supplementary Figure 2B**), suggesting that this alteration is not drug-related. We applied clustering analysis on pre-gated NK cells and identified four cell clusters (**Figure 2E**) that do not differ in frequency between HC and SLE, indicating that there is no NK subpopulation that is characteristic of SLE patients and could be used as a biomarker. Interestingly, we observed that cluster 2 has a CXCR5 expressing subpopulation and cluster 4 one expressing KIR3DL1, which are only present in SLE patients (**Figure 2E**). Further research is warranted to understand the pathophysiological importance of these subpopulations.

### SLE NK Cells Fail to Upregulate SLAMF1 and SLAMF7 in Response to Cytokine Stimulation

**Figure 3:**
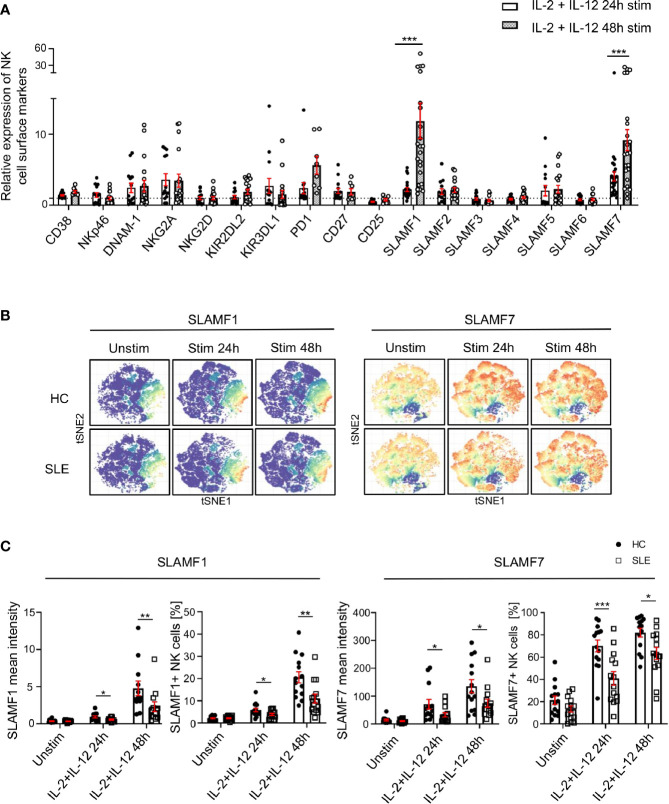
SLAMF1 and SLAMF7 fail to be properly upregulated on the surface of SLE NK cells after activation with cytokines. **(A)** Expression of surface markers after 24h and 48h of stimulation with cytokines on healthy NK cells, standardized to their level of expression on unstimulated cells (HC=23, SLE=23; Mixed-effects analysis with Sidak’s multiple comparison test). **(B)** t-SNE presentation of the expression level of SLAMF1 and SLAMF7 in HC and SLE patients before and after stimulation with cytokines (down-sample HC=12’000 cells, SLE=12’000 cells). **(C)** Comparison of expression of NK cell surface markers after 24h and 48h of stimulation with cytokines between HC and SLE patients as mean intensity (above) and frequency (below) (HC=14, SLE=14; two-way ANOVA and Sidak’s multiple comparison). Data represent mean ± SEM (*P < 0.05, **P < 0.01, ***P < 0.001). HC, healthy controls.

Since the response of SLE NK cells to cytokine stimulation is impaired and considering that the function of NK cells relies on their extracellular phenotype (9), we examined the expression of NK cells surface receptors following stimulation with IL-2 and IL-12 for up to 48h in SLE patients and matched HC. We observed a marked upregulation of SLAMF1 and SLAMF7 on NK cells from HC, 11-fold and 9-fold respectively at 48h of cytokines stimulation, compared to unstimulated cells (**Figure 3A**). Interestingly, PD-1 also shows a 5.6-fold increase at 48h of stimulation (**Figure 3A**). Of note, CD38 is not significantly upregulated after NK cells are activated with cytokines (**Figure 3A**). However, NK cells from SLE patients failed to upregulate certain cell surface receptors to the same extent as HC (**Figures 3B, C**; **Supplementary Figure 3**). More specifically, although SLAMF1 expression is also upregulated on SLE NK cells upon cytokine stimulation, the upregulation is less prominent than that observed in HC (**Figures 3B, C** **)**. Similarly, NK cells from SLE patients fail to upregulate SLAMF7 and PD-1 to the same extent as HC (**Figures 3B, C**; **Supplementary Figure 3**). Overall, our data shows that NK cells from SLE patients fail to adequately upregulate SLAMF1 and SLAMF7 in response to cytokine stimulation.

### Engagement of CD38 and SLAMF7 With Specific Monoclonal Antibodies (mAb) Enhances the Function of Healthy and SLE NK Cells

**Figure 4:**
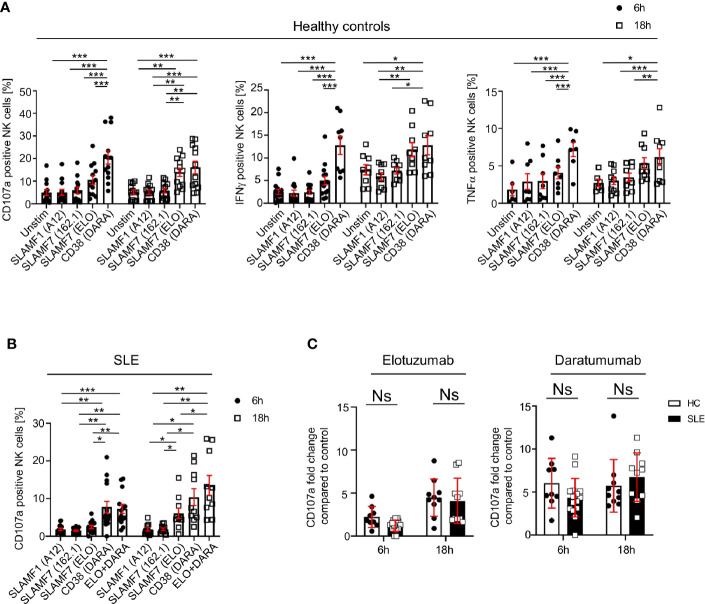
Engagement of SLAMF7 and CD38 with specific mAb enhances the function of healthy and SLE NK cells. **(A)** Degranulation (CD107a) and production of cytokines (IFNγ and TNFα) in NK cells of healthy controls after stimulation with daratumumab (N=14) and elotuzumab (N=12, Mixed-effect analysis with Tukey’s multiple comparison test). **(B)** Degranulation (CD107a+) in NK cells of SLE patients after stimulation with daratumumab (6h N=15, 18h N=10) and elotuzumab (6h N=14, 18h N=10; two-way ANOVA analysis and Sidak’s multiple comparison test). **(C)** Fold change of CD107a compared to control after 6h or 18h stimulation with elotuzumab or daratumumab in HC and SLE patients (HC 6h=9, HC 18h=10, SLE 6h=15, SLE 18h=10; mixed-effect analysis with Sidak’s multiple comparison). Data represent mean ± SEM (*P < 0.05, **P < 0.01, ***P < 0.001). HC, healthy controls.

We investigated how the engagement of SLAMF1, SLAMF7 and CD38 with mAb influences NK cell function, by examining the production of cytokines, degranulation and cell viability after 6h and 18h. Ligation with elotuzumab, a humanized anti-SLAMF7 mAb approved to treat relapsing multiple myeloma (15), promotes NK cells degranulation and IFNγ production after 18h, whereas no significant NK cells activation was observed at 6h of stimulation in HC (**Figure 4A**). Another clone of anti-SLAMF7 mAb (clone 162.1), which has been shown to enhance the cytotoxic response of SLE CD8+ T cells in response to viral antigen (16), did not produce any significant effect on NK cells degranulation (**Figure 4A**). NK cells stimulation with daratumumab, a mAb that agonizes CD38, strongly enhanced NK cells degranulation, IFNγ and TNFα production (**Figure 4A**). Interestingly, in healthy controls daratumumab effectively promotes NK cell degranulation and production of IFNγ and TNFα after 6h of stimulation, whereas elotuzumab takes longer to activate NK cells (18h) and only promotes degranulation and production of IFNγ, but not TNFα. Stimulation of NK cells with anti-SLAMF1 mAb (clone A12) did not result in any effect on degranulation or cytokine production (**Figure 4A**).

Next, we examined the effect of SLAMF7 ligation with elotuzumab and of CD38 with daratumumab on NK cells from SLE patients. Based on our results from healthy controls, we used anti-SLAMF1 (clone A12) as negative control. We observed that in SLE NK cells both daratumumab and elotuzumab promote degranulation, after 6h and 18h respectively, to the same extent as in HC (**Figures 4B, C** **)**. However, compared to results obtained in healthy controls, daratumumab and elotuzumab do not promote cytokine production by SLE NK cells (**Supplementary Figure 4A**). Furthermore, the magnitude of degranulation at 18h is, for both HC and SLE NK cells, more prominent following ligation with daratumumab (6-fold) compared to elotuzumab (4-fold) (**Figure 4C**). In addition, we examined NK cells viability after stimulation with elotuzumab and daratumumab. Both antibodies lead to a slight increase in mortality of NK cells compared to the control condition (**Supplementary Figure 4B**). Eventually, we examined the effect of elotuzumab and daratumumab on other lymphocyte subsets and observed no effect on the viability or activation of CD4+, CD8+ T cells and B cells (**Supplementary Figures 5** and **6**).

Altogether, our data shows that elotuzumab and daratumumab specifically activate SLE NK cells by promoting their cytotoxic activity.

### Expression of CD38, SLAMF1, and SLAMF7 Characterizes SLE Circulating Plasma Cells

**Figure 5:**
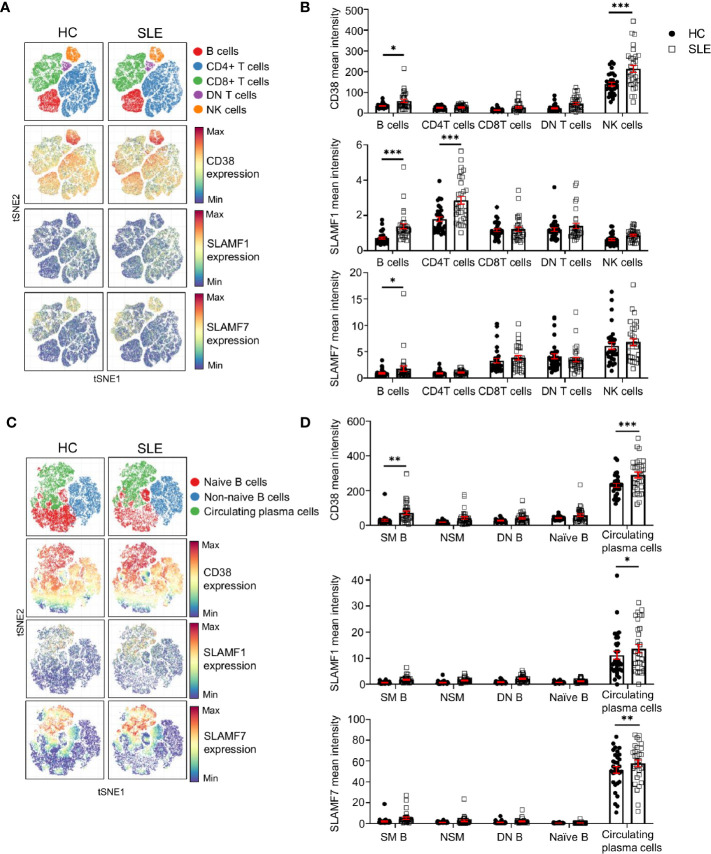
Expression of CD38, SLAMF1 and SLAMF7 characterizes SLE circulating plasma cells. ***(*A*)*** t-SNE presentation of the expression level of CD38, SLAMF1 and SLAMF7 in main lymphocyte populations for HC and SLE patients (down-sample HC=100’000 cells and SLE=100’000 cells). **(B)** Comparison of mean expression level of CD38, SLAMF1 and SLAMF7 between HC and SLE patients in main lymphocyte populations (HC and SLE=31; two-way ANOVA with Sidak’s multiple comparison test). **(C)** t-SNE presentation of the expression level of CD38, SLAMF1 and SLAMF7 in B cell subpopulations for HC and SLE patients (down-sample N=10’000 cells per subpopulation HC and SLE=26). **(D)** Comparison of the mean expression level of CD38, SLAMF1 and SLAMF7 between HC and SLE patients in B cell sub-populations (HC=31, SLE =31; two-way ANOVA with Sidak’s multiple comparison test, SM, switch memory; NSM, non switch memory; DN, double negative). Data represent mean ± SEM (*P < 0.05, **P < 0.01, ***P < 0.001). HC, healthy controls.

The above-mentioned cell surface receptors are important in cell-to-cell contact. Therefore, to understand their relevance for the pathophysiology of SLE, we investigated their expression on other major lymphocyte populations. We manually gated on CD4+, CD8+, DN T cells (CD4- CD8- double negative T cells), B cells and NK cell. We then exploited t-SNE analysis to visualize the expression of CD38, SLAMF1 and SLAMF7 on these cell populations (**Figure 5A**) and quantified their relative expression levels in SLE patients and HC (**Figure 5B**).

Other than NK cells, our data indicates that all three receptors are expressed at higher levels on SLE B cells compared to HC. CD38 expression did not show any difference in its expression between SLE and HC in any other lymphocyte population included in this study (**Figures 5A, B** **)**. The expression of SLAMF1 is significantly higher on B cells and on CD4+ T cells from SLE patients as previously described (17). In addition, our data shows that SLAMF7 is increased on total B cells from SLE patients, despite a low expression level compared to other lymphocytes such as NK cells, CD8+ and DN T cells. We further examined the expression of these receptors on B cell subpopulations. A t-SNE analysis of naïve B cells (CD19+ CD27- IgD+), non-naïve B cells (CD19+ which are not CD27- IgD+) and circulating plasma cells (CD19+ CD20- CD27+ CD38+ IgD-), showed that all three molecules are expressed at a higher level on circulating plasma cells compared to other B cell subpopulations (**Figures 5C, D** **)**. Moreover, the level of expression of all the three receptors is increased in SLE circulating plasma cells compared to HC, suggesting that these molecules could contribute to the dysfunction of SLE B cells.

### Activation of SLE NK Cells With mAb Directed Against CD38 and SLAMF7 Promotes the Killing of Peripheral Blood Plasma Cells

We evaluated whether the activation of NK cells can promote the killing of SLE peripheral blood plasma cells. We generated a NK-B cell *in vitro* co-culture system, in which we pre-stimulated NK cells of HC with elotuzumab or daratumumab, then co-cultured them with autologous B cells and measured the mortality of B cell subsets.

**Figure 6:**
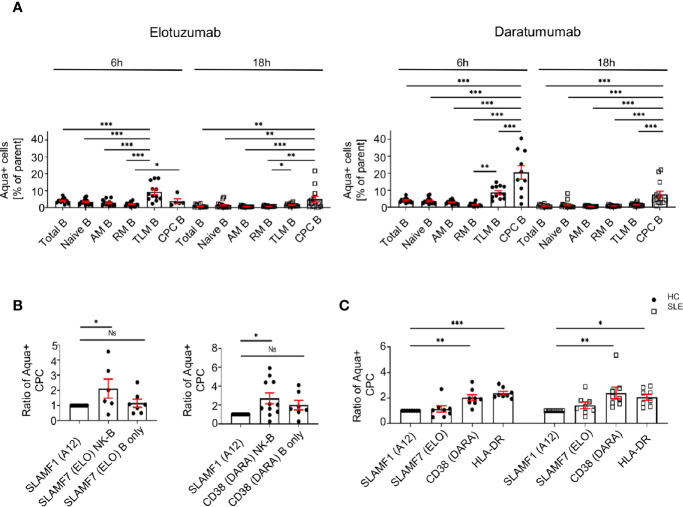
Activation of SLE NK cells with mAb directed against CD38 and SLAMF7 promotes the killing of peripheral blood plasma cells. **(A)** Frequency of dead cells in a NK-B cell co-culture system after 6h (N=14) and 18h (N=16) following stimulation with SLAMF1, elotuzumab or daratumumab (one-way ANOVA with Tukey’s multiple comparison test). **(B)** Fold increase of dead circulating plasma cells following stimulation with daratumumab 6h (N=12) or elotuzumab 18h (N=7) in either B cells alone or B cells co-cultured with pre-stimulated NK cells (one-way ANOVA with Sidak’s multiple comparison test) **(C)** Ratio of dead cells over control condition after 6h stimulation with elotuzumab or daratumumab in HC and SLE patients (HC=8, SLE=8; two-way ANOVA, Sidak’s multiple comparison test). Data represent mean ± SEM (*P < 0.05, **P < 0.01, ***P < 0.001). HC=healthy controls.

First, our data shows that elotuzumab (18h) and daratumumab (6h) can efficiently kill circulating plasma cells, leading to 2.1 and 2.7 fold more dead cells compared to negative control (SLAMF1 stimulation) respectively (**Figure 6A**). Furthermore, when NK cells are activated with either mAb they kill circulating plasma cells specifically, sparing other B cell subpopulations, such as naïve, activated, resting and tissue like memory cells (**Figure 6A**). Second, we observed that the presence of NK cells is necessary to achieve significant killing of circulating plasma cells, although both mAb alone have a minor impact on circulating plasma cell mortality (**Figure 6B**).

Due to the restrictions in the SLE sample size that we can obtain, we could not repeat this assay in SLE patients. Accordingly, we isolated CD3- cells and stimulated them with mAb. We observed that at 6h, treatment with daratumumab significantly killed circulating plasma cells of SLE patients to the same extent as in the matched HC (**Figure 6C**). In conclusion, our results strongly suggest that these mAb act through the activation of SLE NK cells and effectively kill SLE circulating plasma cells.

## Discussion

We exploited single-cell mass cytometry to decipher the phenotypic alterations that characterize SLE NK cells. Our data identified CD38 as being highly expressed on SLE NK cells compared to HC. Moreover, we observed that SLE NK cells fail to properly upregulate SLAMF1 and SLAMF7 when activated with cytokines; two receptors that play an important role in cell-to-cell interaction. We showed that these three receptors are also highly expressed on SLE peripheral blood plasma cells, a cell population that contributes to the production of autoantibodies in SLE. In addition, we demonstrated that mAb directed against CD38 and SLAMF7 receptors enhance the degranulation of SLE NK cells and selectively promote the killing of peripheral blood plasma cells. Overall, our data suggests that the dysregulation of SLAMF1 and SLAMF7 on the surface of SLE NK cells contribute to their dysfunction and might impair their interaction with plasma cells, resulting in an accumulation of autoantibody producing cells. Additionally, targeting NK cells with activating mAb may represent an attractive direction to eliminate autoantibody-producing cells in SLE.

SLAMF1 and SLAMF7 belong to the signaling lymphocytic activation molecule family receptors. A unique feature of these two SLAMF members is that they act as self-ligand (18). The involvement of SLAMF molecules in SLE pathogenesis has been repeatedly reported (16–24) as well as their importance in NK cells activation and interaction with other cell types (25, 26). SLAMF1 has been reported to be expressed at a higher level on SLE B cells and CD4+ T cells compared to their healthy counterparts and its importance for SLE B cell function was previously reported (26). However, its potential role on SLE NK cells was not previously described. SLAMF7 has been shown to be highly expressed by cytotoxic cells and plasma cells. SLAMF7 displays an altered expression, function and/or regulation on SLE NK cells and CD8+ T cells (16, 19, 22), supporting a role of this molecule in SLE pathogenesis. The importance of SLAMF7 was described in multiple myeloma, where elotuzumab was approved to treat disease relapse (15). The binding of elotuzumab contributes to the elimination of myeloma cells, through various mechanisms including the activation of NK cells cytotoxic response and antibody dependent cell-mediated cytotoxicity (27). A previous study has shown that the ligation of SLAMF7 in SLE promotes the degranulation of CD8+ T cells in response to viral antigens, therefore empowering the antiviral response that is compromised in patients with SLE (16), highlighting the potential therapeutic benefit of targeting SLAMF7.

CD38 is a surface glycoprotein with ectoenzymatic functions and is expressed at high levels on plasma cells. Like SLAMF7, CD38 has been identified as a target for mAb to eliminate myeloma cells in patients with relapsing multiple myeloma with the use of anti-CD38 daratumumab (28). A recent report has shown that daratumumab represents a potential therapeutic approach to eliminate antibody-producing plasma cells in SLE patients (29). Furthermore, it has been shown to ameliorate clinical manifestations and to eliminate antibody producing plasma cells in two patients with refractory SLE (30). A subset of SLE patients who are highly susceptible to infections, exhibit an altered CD8+ T cells cytotoxic response and express a high level of CD38 on their surface (31), thus further underlining the potential benefits of targeting CD38. Our data stresses a preponderant role of NK cells in the process leading to plasma cell depletion by daratumumab, as stimulation of isolated NK cells with daratumumab is sufficient to promote the killing of circulating plasma cells in culture, whilst the sole exposure of B cells to the drug isn’t.

As previously reported, PD-1 is also upregulated on NK cells in response to inflammatory cytokines (32). This increase is significantly altered in SLE NK cells and likely reflects SLE NK cells compromised activation status. Further investigation is warranted on this aspect.

Our study reveals interesting differences between daratumumab and elotuzumab. Elotuzumab promotes degranulation of NK cells and IFNγ production, but not the production of TNFα by NK cells. Since elevated TNFα levels have been described in SLE patients and may contribute to the pathogenesis of organ damage (33), this property could be of interest if elotuzumab was to be considered as a therapeutic option in SLE. On the other hand, SLE NK cell degranulation and elimination of antibody-producing cells *in vitro* is more robust when NK cells are activated with daratumumab compared to elotuzumab.

Our study has several limitations. First, the use of single-cell mass cytometry limits the identification of cell surface receptors to the antibodies included in our panels. Compared to RNA seq, this method monitors fewer targets but directly identifies cell surface proteins that can be targeted by therapeutic mAb. Second, further experiments are warranted to identify the individual implications of the three cell surface markers evaluated in this study in the interaction between NK cells and circulating plasma cells. So far, this aspect remains unexplored due to the limited number of circulating plasma cells available from the peripheral blood of patients and controls. We are working on plasma cell line culture system that will allow to individually silence each receptor. Finally, examination of secondary lymphoid organ and bone marrow aspirations would allow examination of B cells during their maturation process and long-lived plasma cells. However, these tissues are difficult to obtain.

In conclusion, the failure of SLAMF1 and SLAMF7 regulation on SLE NK cells might contribute to an impaired interaction between NK cells and plasma cells. This might lead to the accumulation of antibody producing plasma cells that characterizes SLE. From this point of view, restoration of NK cell cytotoxicity may contribute to the elimination of SLE plasma cells. Targeting SLAMF7 with elotuzumab and CD38 with daratumumab contributes to the elimination of antibody producing cells *in vitro* and this elimination occurs, at least in part, through the restoration of SLE NK cells degranulation. Because both elotuzumab and daratumumab are safe when used to treat multiple myeloma and appear to be well-tolerated when administrated to SLE patients, their utilization should be evaluated in controlled studies to assess their efficacy to treat SLE.

## Data Availability Statement

The raw data supporting the conclusions of this article will be made available by the authors, without undue reservation.

## Ethics Statement

The studies involving human participants were reviewed and approved by Commission cantonale d’Ethique de la Recherche sur l’être humain CER-VD (SwissEthics 2017-01434). The patients/participants provided their written informed consent to participate in this study.

## Author Contributions

DC: study design and data analysis. MH, FB, and NF: conducting experiments, data acquisition and analysis. CR and AH: recruitment of SLE patients and healthy controls. CF: responsible of CyTOF facility. MH and MS: bioinformatics analysis. DC and MH: writing and editing of manuscript. All authors contributed to the article and approved the submitted version.

## Funding

This work was funded by a grant from the Swiss National Science Foundation Ambizione PZ00P3_173950 (to DC) and a grant from the Novartis Foundation (to DC).

## Conflict of Interest

The authors declare that the research was conducted in the absence of any commercial or financial relationships that could be construed as a potential conflict of interest.
