# Supplementary material for: Restoration of NK Cell Cytotoxic Function With Elotuzumab and Daratumumab Promotes Elimination of Circulating Plasma Cells in Patients With SLE
Source: Front Immunol. 2021 Mar 22;12:645478. doi: 10.3389/fimmu.2021.645478 (PMC8019934; doi:10.3389/fimmu.2021.645478)
Supplement: Supplementary file 1 [file Table_1.docx]

Supplementary Material

**Supplementary Table 1.** Antibody list

| Flow Cytometry antibody | Format | Clone | Company |
| --- | --- | --- | --- |
| Anti-CD3 | BUV737 | UCHT1 | BD bioscience |
| Anti-CD4 | Pacific Blue | RPA-T4 | BD bioscience |
| Anti-CD8 | BV605 | SK1 | BD bioscience |
| Anti-CD19 | FITC | REA675 | Miltenyi |
| Anti-CD56 | BUV395 | NCAM16.2 | BD bioscience |
| Anti-CD20 | Pacific Blue | 2H7 | Biolegend |
| Anti-CD21 | AlexaFluor700 | Bu32 | Biolegend |
| Anti-CD24 | APC-eFluor780 | eBioSN3 | invitrogen |
| Anti-CD38 | PE-CF594(ECD ) | HIT2 | BD bioscience |
| Anti-SLAMF7 | PE | 162.1 | Biolegend |
| Anti-CD27 | AlexaFluor700 | O323 | Biolegend |
| Anti-Annexin V | APC | - | BD bioscience |
| Anti-TNFα | APC | - | BD bioscience |
| Anti-IFNγ | AlexaFluor700 | B27 | BD bioscience |
| Anti-CD107a | PE | H4A3 | BD Pharmingen |
| Anti-CD27 | PE-Cy7 | O323 | ebioscience |
|  |  |  |  |
| **Mass Cytometry Antibody** | **Format** | **Clone** | **Company** |
| CD45 | 89Y | HI30 | Fludigm |
| Live/Dead | 103Rh | - | Fludigm |
| CD8 | 113 In | RPA-T8 | Biolegend |
| CD4 | 115 In | RPA-T4 | Biolegend |
| CD45 | 139 La | HI30 | Conju-Biolegend |
| CD196/CCR6 | 141 Pr | 11A9 | Fludigm |
| CD19 | 142 Nd | HIB19 | Fludigm |
| CD352 / SLAM 6 | 143 Nd | NT-7 | Fludigm |
| CD38 | 144 Nd | HIT2 | Biolegend |
| CD127 | 145 Nd | A019D5 | Biolegend |
| IgD | 146 Nd | IA6-2 | BD bioscience |
| CD7 | 147 Sm | CD7-6B7 | Fludigm |
| CD45 | 148 Nd | HI30 | Conju-Biolegend |
| CCR4 | 149 Sm | 205410 | Fludigm |
| CD3 | 150 Nd | UCH-T1 | BD bioscience |
| CD123 | 151 Eu | 6H6 | Fludigm |
| PD-1 | 151 Eu | EH12.2H7 | Biolegend |
| CD21 | 152 Sm | BL13 | Fludigm |
| CD45RA | 153 Eu | HI100 | BD bioscience |
| CD84 / SLAM 5 | 154 Sm | CD84.1.21 | Fludigm |
| CD27 | 155 Gd | L128 | Fludigm |
| SLAMF 7 (CD319) | 156 Gd | 162.1 | Biolegend |
| CXCR3 | 158 Gd | 1C6/CXCR3 | BD bioscience |
| CCR7 | 159 Tb | G043H7 | Biolegend |
| CD337 / NKp30 | 159 Tb | Z25 | Fludigm |
| CD14 | 160 Gd | M5E2 | Fludigm |
| CD150 / SLAM 1 | 161 Dy | A12(7D4) | Biolegend |
| CD11c | 162 Dy | clone 3.9 | Fludigm |
| CD335 /NKp46 | 162 Dy | BAB281 | Fludigm |
| CRTh2 (Fluidigm) | 163 Dy | BM16 | Fludigm |
| CD48 / SLAM 2 | 164 Dy | BJ40 | Biolegend |
| CD45RO | 165 Ho | UCHL1 | Fludigm |
| CD45 | 166 Er | HI30 | Conju-Biolegend |
| NKG2D | 166 Er | ON72 | Fludigm |
| CXCR5 | 167 Er | RF8B2 | Biolegend |
| CD158e1 (KIR3DL1/NKB1) | 167 Er | DX9 | Fludigm |
| ICOS | 168 Er | C398.4A | Biolegend |
| CD25 | 169 Tm | 2A3 | Fludigm |
| CD159a (NKG2a) | 169 Tm | HI100 | Fludigm |
| TCR va24-Ja18 (6B11) | 170 Er | Witek | Fludigm |
| CD226 / DNAM-1 | 171 Yb | DX11 | Fludigm |
| CD20 | 171Yb | 2H7 | Fludigm |
| TCRαβ | 172 Yb | IP26 | Biolegend |
| HLA-DR | 173 Yb | L243 | Fludigm |
| CD158b (KIR2DL2/L3/NKAT2) | 173 Yb | DX27 | Fludigm |
| CD229 / SLAM 3 | 174 Yb | HLy9.1.25 | Fludigm |
| CD244 / SLAM 4 | 175Lu | C1.7 | Biolegend |
| CD56 | 176Yb | R19-760 | Fludigm |
| CD57 (CHUV) | 194 Pt | NK1 | Conju-BD bioscience |
| CD45 | 194 Pt | HI30 | Conju-Biolegend |
| CD45 | 198 Pt | HI30 | Conju-Biolegend |
| CD16 | 209Bi | 3G8 | Fludigm |
|  |  |  |  |
| **Purified Antibody** | **Format** | **Clone** | **Company** |
| Anti-SLAMF1 (CD150) | Purified | A12(7D4) | Biolegend |
| Anti-SLAMF7 (CD319) | Purified | 162.1 | Biolegend |
| Anti-SLAMF7 (CD319) | - | Elotuzumab | BMS |
| Anti-CD38 | - | Daratumumab | Janssen-Cilag AG |

**
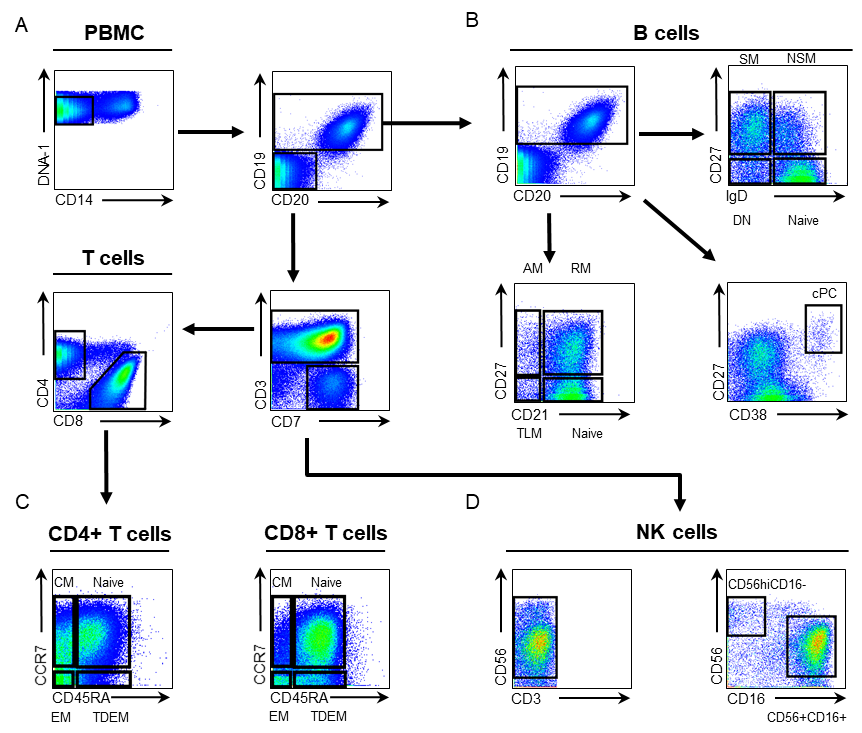
**

**Supplementary Figure 1. Single-cell mass cytometry gating strategy.** (A) Following debarcoding, removal of doublets and gating on live cells, PBMCs were gated as shown in this representative example and as mentioned in specific experiments. (B) B cell differentiated populations are defined as follows: switched memory (SM) CD27+IgD-; naïve B cells CD27-IgD+ or CD27-CD21+; non-switched memory (NSM) CD27-IgD+; double negative CD27-IgD-; activated memory (AM) CD27+CD21-; resting memory (RM) CD27+CD21+; tissue-like memory (TLM) CD27-CD21-; circulating plasma cells (cPC) CD27+CD38+. (C) CD4+ and CD8+ differentiated T cells are defined as follows: central memory (CM) CCR7+CD45RA-; naïve T cells CCR7+CD45RA+; terminally differentiated effector memory (TDEM) CCR7-CD45RA+; effector memory (EM) CCR7-CD45RA-. (D) NK cells subpopulations are CD56hiCD16- and CD56+CD16+.

**
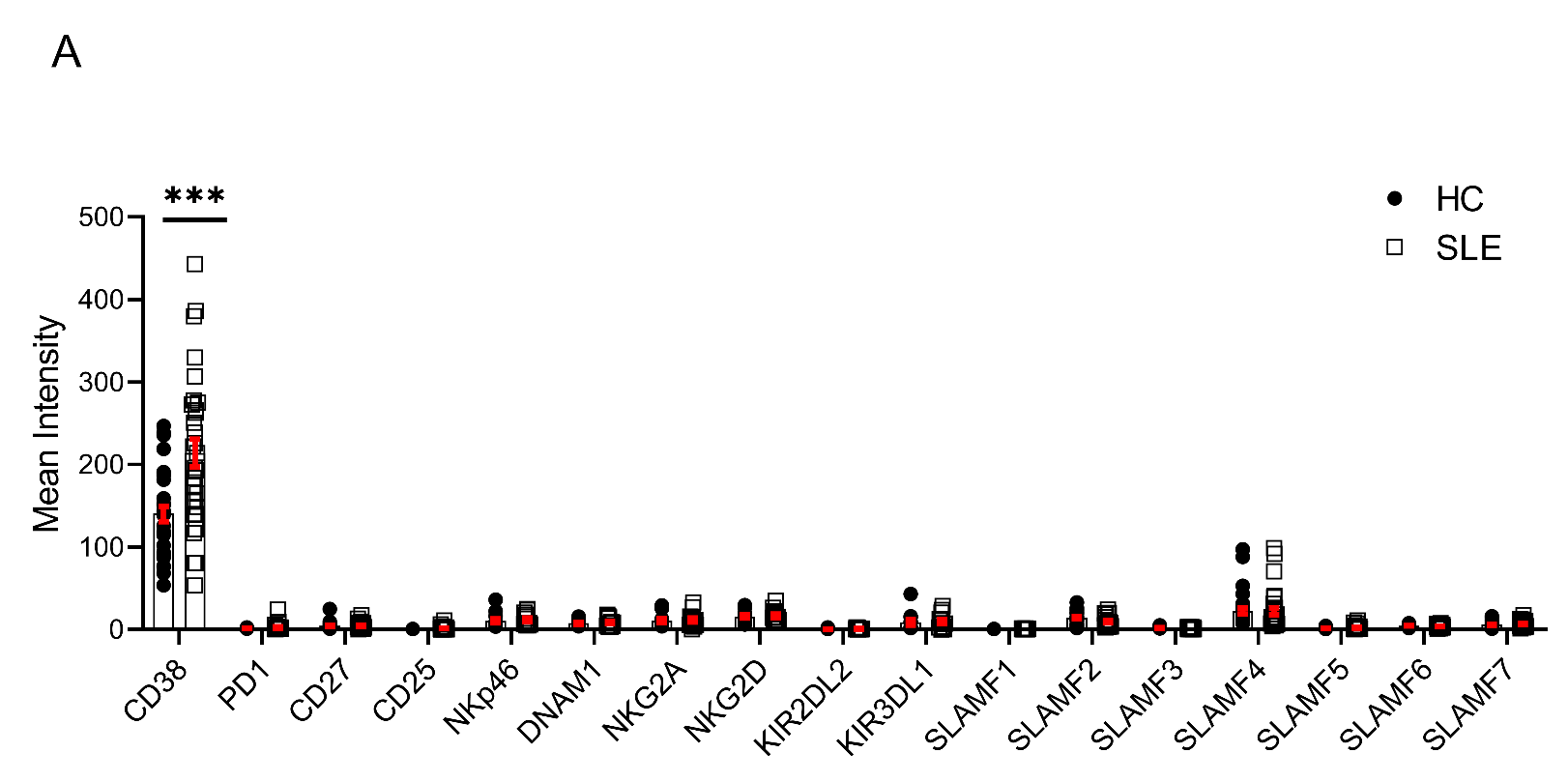

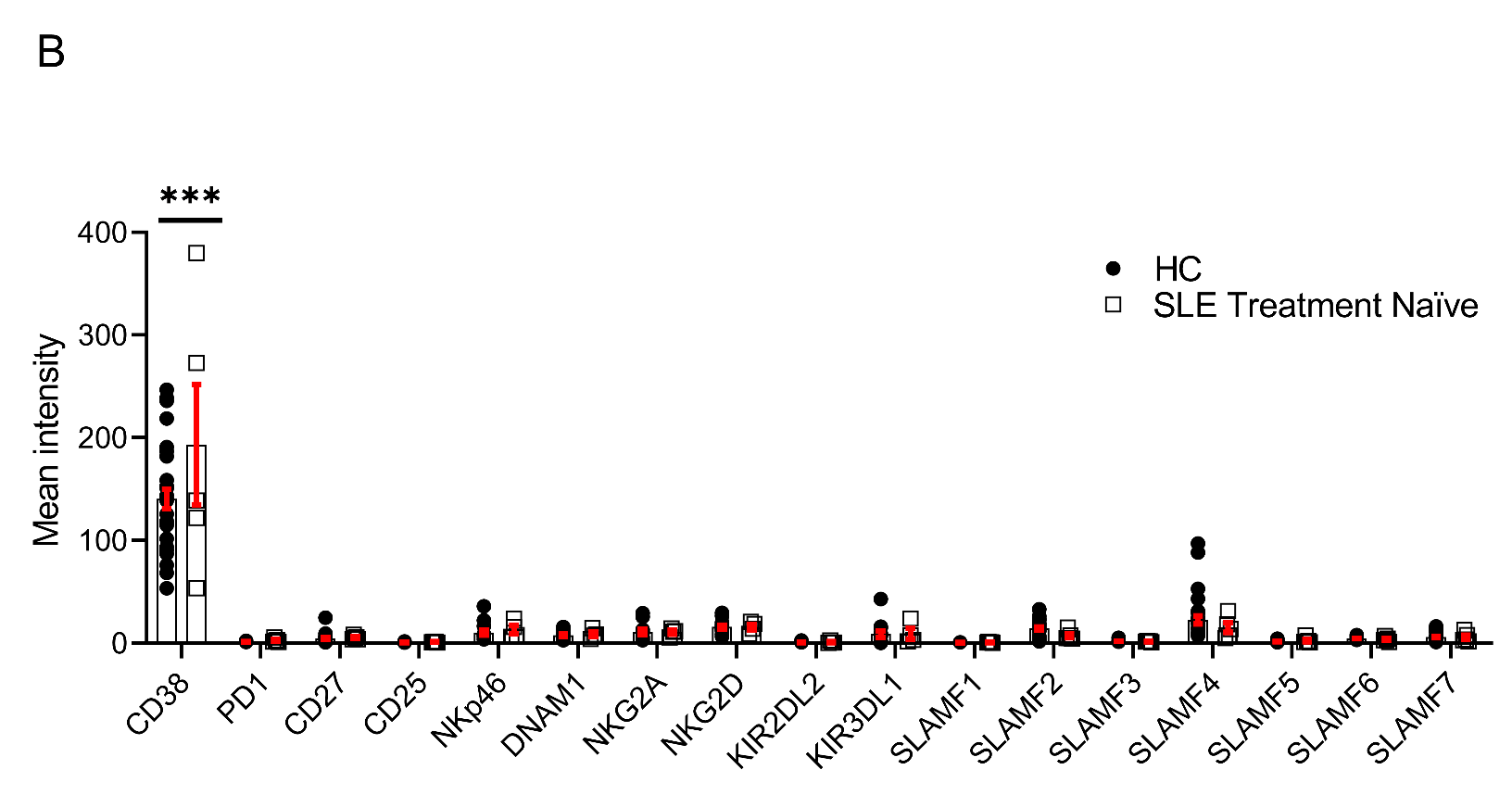
**

**Supplementary Figure 2. Analysis of NK cell surface markers in HC and SLE patients by single-cell mass cytometry.** Mean intensity of indicated cell surface markers expressed on NK cells in HC vs (A) SLE patients and (B) treatment-naïve SLE patients (HC=32, SLE=32, treatment naïve SLE=7; two-way ANOVA and Sidak’s multiple comparison test; ***P<0.001). Data represent mean ± SEM. HC=healthy controls.

**
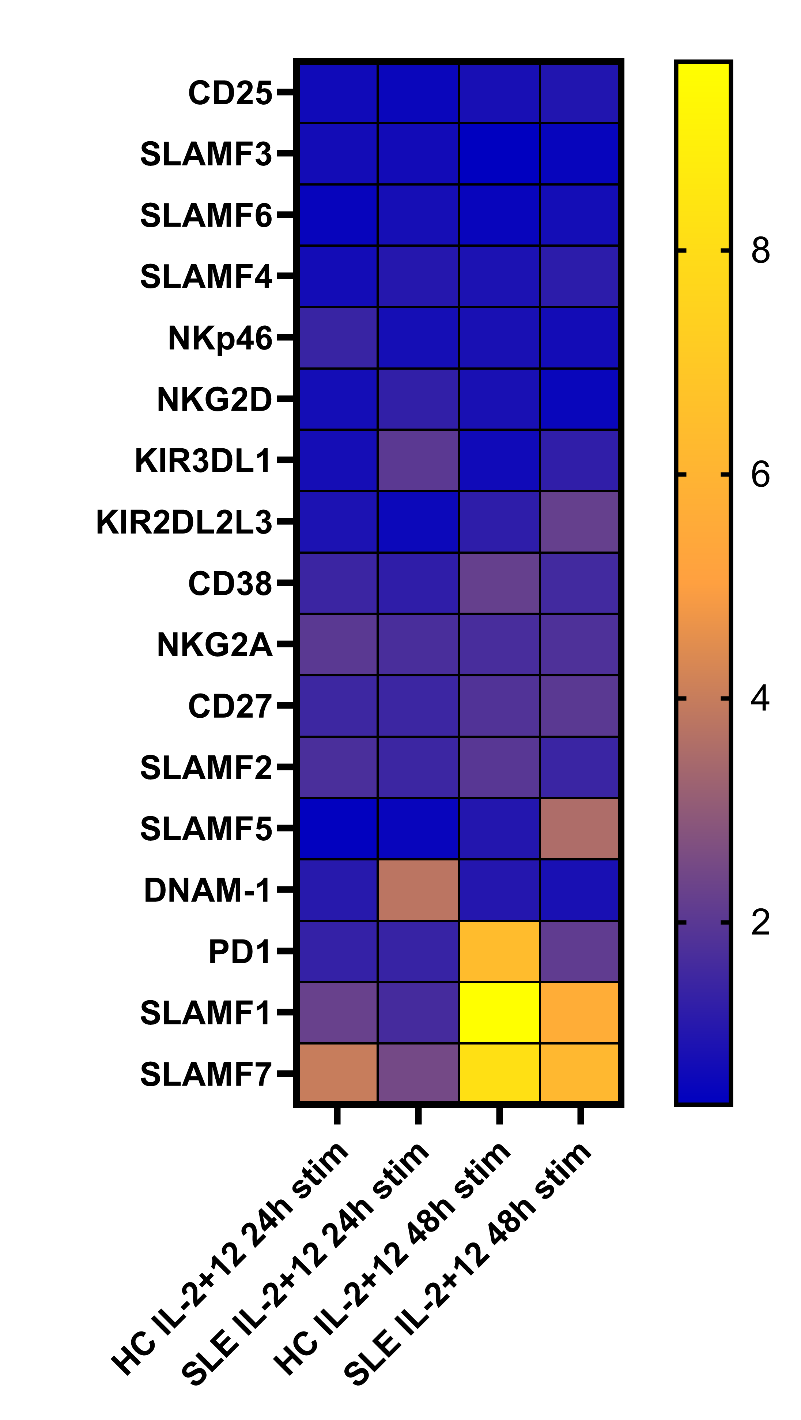
**

**Supplementary Figure 3. NK surface receptor alteration after stimulation in HC and SLE patients.** Heatmap showing the relative of expression of markers in HC and SLE patients at 24h and 48h of stimulation with IL-2+IL-12 (HC=12, SLE=12). Expression is normalized to an unstimulated condition. HC=healthy controls.

**Supplementary Figure 4. Effect of elotuzumab and daratumumab on cytokine production and viability in NK cells.** (A) Cytokine production in SLE NK cells (IFNg and TNFa) after 6h and 18h of stimulation with elotuzumab and/or daratumumab (6h N=14, 18h N=10; two-way ANOVA analysis with Sidak’s multiple comparison). (B) Effect of elotuzumab and daratumumab on the viability of NK cells at 6h and 18h of stimulation (N=6; two way ANOVA with Sidak’s multiple comparison). Data represent mean ± SEM. HC=healthy controls.

**Supplementary Figure 5. Effect of elotuzumab and daratumumab on cell viability in different lymphocyte populations of HC and SLE patients.** Effect of elotuzumab and daratumumab on CD4+ T, CD8+ T and CD19+ cells viability at 6h and 18h of stimulation. Data represent mean ± SEM. HC=healthy controls.

**Supplementary Figure 6. Effect of elotuzumab and daratumumab on cytokine production by different lymphocyte populations of HC and SLE patients.** Effect of elotuzumab and daratumumab on degranulation and/or cytokine production by CD4+ T, CD8+ T and B cells. Data represent mean ± SEM. HC=healthy controls.
